# Supplementary material for: Human Health Risk Assessment for Exposure to Heavy Metals via Dietary Intake of Rainbow Trout in the Influence Area of a Smelting Facility Located in Peru
Source: Toxics. 2023 Sep 8;11(9):764. doi: 10.3390/toxics11090764 (PMC10535794; doi:10.3390/toxics11090764)
Supplement: Supplementary file 1 [file toxics-11-00764-s001.zip › toxics-2550843-SI.pdf]

Table S1. Descriptive statistics of bioconcentration factor of heavy metals and As.

Variable	Sector	SD	Min	Median	Max
BCF-Cu	Lower course	6.72	1.97	10.77	21.91
	Middle course	7.16	2.41	9.97	24.83
	Upper course	5.08	4.12	7.55	20.06
BCF-Pb	Lower course	5.54	2.79	6.42	21.00
	Middle course	2.37	3.04	6.67	10.48
	Upper course	4.21	4.44	9.35	18.77
BCF-Zn	Lower course	16.97	21.47	40.57	82.28
	Middle course	21.95	22.71	37.90	95.96
	Upper course	19.82	23.57	40.87	80.91
BCF-As	Lower course	0.55	0.36	1.12	2.22
	Middle course	0.63	0.87	1.44	3.25
	Upper course	1.25	0.70	3.29	4.46

Table S2. Descriptive statistics of biosediment accumulation factor of heavy metals and As.

Variable	Sector	SD	Min	Median	Max
BSAF-Cu	Upper course	0.0022	0.0062	0.0097	0.0146
	Middle course	0.0062	0.0075	0.0127	0.0291
	Lower course	0.0031	0.0042	0.0109	0.0142
BSAF-Pb	Upper course	0.0018	0.0027	0.0050	0.0086
	Middle course	0.0019	0.0043	0.0064	0.0101
	Lower course	0.0015	0.0019	0.0045	0.0068
BSAF-Zn	Upper course	0.0024	0.0114	0.0140	0.0185
	Middle course	0.0014	0.0133	0.0154	0.0177
	Lower course	0.0018	0.0085	0.0113	0.0138
BSAF-As	Upper course	0.0001	0.0001	0.0003	0.0004
	Middle course	0.0002	0.0003	0.0006	0.0010
	Lower course	0.0009	0.0008	0.0026	0.0032
